# Olfactory stimulation for people with dementia: A rapid review

**DOI:** 10.1177/14713012221082377

**Published:** 2022-04-17

**Authors:** Federica D’Andrea, Victoria Tischler, Tom Dening, Anne Churchill

**Affiliations:** School of Biomedical Sciences, 7364 University of West London, London, UK; School of Biomedical Sciences, 7364 University of West London, London, UK; 3286 University of Exeter, Exeter, UK; Institute of Mental Health & School of Medicine, 6123 University of Nottingham, Nottingham, UK; 316493 Givaudan UK Ltd, Ashford, UK

**Keywords:** Alzheimer’s disease, aromatherapy, olfactory stimuli, olfaction, psychosocial intervention

## Abstract

There is a growing interest in using olfactory (smell) stimulation in dementia care. This study aims to extend current knowledge by synthesising the evidence on the efficacy of interventions using olfactory stimulation for people with dementia and to assess the effects of different types of odours and administration methods using a mixed methods approach. The rapid review was conducted based on searches in six electronic databases. A narrative approach was applied to assess 20 studies included in the review. Fourteen studies used a quasi-experimental design, five studies used an experimental design and one was a case study. High heterogeneity was found on odours and methods of application used, with the majority of studies administering lavender oil using a diffuser. Mixed results were reported on the benefits of olfactory stimulation on responsive behaviours and cognitive function. Although the evidence available is limited, encouraging results were found regarding olfactory stimulation and increased sleep duration, food intake and improved balance. It was not possible to draw any overall conclusion in relation to the effect of olfactory stimulation. However, this review shows promising results that support further investigation of olfactory stimulation as a nonpharmacological intervention for people with dementia. The review is limited due to the low to moderate quality of studies included. Furthermore, the broad range of approaches was employed, and comparison between the studies was difficult. Further high-quality mixed method studies using robust and detailed protocols are needed to clarify the effects of olfactory stimuli and any other factors that may influence the responses of people with dementia.

## Introduction

Dementia is increasingly recognised as a global healthcare challenge (World Health Organization, 2020). Reduced sense of smell has been reported in individuals with dementia (Murphy, 2019). Studies have found that changes in olfaction occur in the early stage of the disease and sometimes even before the person manifests the onset of clinical symptomatology (e.g. Attems et al., 2014; Murphy, 2019). The sense of smell plays an important role in everyday life. It enables humans to perceive odours in our surroundings such as flowers, clean laundry and personal care products and serves as a first warning signal, detecting smoke from a fire, leaking natural gas or spoiled food. This powerful sense also mediates flavours of foods and drinks (Doty & Kamath, 2014). Therefore, olfactory impairment or disorders can have a significant negative impact on individual nutrition, appetite, safety and overall quality of life and well-being.

In the absence of a cure, olfactory stimulation has received increasing interest in dementia care. Various interventions have been used to stimulate the olfactory sense of people living with dementia using a variety of smell-based stimuli. These may include household items such as soap, as well as essential (or natural) oils and fragrance (or synthetic) oils. The most popular olfactory intervention is aromatherapy, using a range of essential oils directly applied to the skin surface or inhaled using, for example, a diffuser or vaporiser (Sowndhararajan & Kim, 2016).

There is evidence that exposure to odours can trigger memories of personal past experiences (or autobiographic memory) and positive emotions (Herz, 1998; Willander & Larsson, 2007). These findings are supported by neurological evidence reporting an activation of areas associated with memories and emotional processes including the amygdala, hippocampus, temporal gyrus and temporal pole during odour exposure (Arshamian et al., 2013; Chu & Downes, 2000; Herz et al., 2004).

Other studies suggest that the olfactory sense may link to implicit memory, which can remain intact in people with dementia (Degel et al., 2001; Degel & Köster, 1999; Fleischman et al., 2005). Implicit memory refers to previous experiences unconsciously influencing later behaviour without conscious awareness (White et al., 2015). This means that implicit odour memory may influence behaviours (e.g. food intake or craving for cigarettes) or mood (e.g. reduction in anxiety or depression) (Herz, 2016). Other evidence suggests that the constituents of essential oils may influence behaviour and alter mood states through the central nervous or endocrine systems (Arruda et al., 2012). For instance, the key constituents of lavender oil, for example, linalyl acetate and linalool are associated with sedative and calming effects (Lis-Balchin & Hart, 1999).

Several studies have reviewed the effects of aromatherapy on a variety of outcomes concerning people with dementia over the last decade (e.g. Hui et al., 2021). A Cochrane systematic review (Ball et al., 2020) evaluated the efficacy and safety of aromatherapy for people with dementia. Two other previous systematic reviews assessed the impact of aromatherapy on managing agitation (Kim et al., 2019) and responsive behaviours and cognitive function (Fung et al., 2012) in individuals with dementia.

In the context of the present review, it is pertinent to note that all reviews included only studies with randomised controlled trial (RCT) design. Although quantitative evidence is informative regarding efficacy in relation to defined outcomes, the findings from qualitative or mixed methods studies provide in-depth understanding of the study’s conclusions by incorporating individuals’ experiences.

Furthermore, three reviews included aromatherapy administered by massage or touch. Although there is limited evidence, studies on touch have reported benefits of massage practice by itself, that is, without olfactory stimuli (Hansen et al., 2006). This suggests that any positive findings from studies applying olfactory stimuli by massage or touch might not be the result of the scent, but the effect of a tactile stimulation or of their interaction.

Building on the reviews of aromatherapy, this study used a mixed methods approach to synthesise the evidence on olfactory stimulation in dementia care by excluding those interventions combining olfactory elements with other activities such as massage. In particular, this review seeks to (1) synthesise the qualitative and quantitative evidence on the impact of olfactory stimulation on responsive behaviours, cognitive function, communication, quality of life, pain and physical functioning; (2) assess the effects of different types of scents used and identify, if any, patterns in their effects and (3) review the different ways in which olfactory stimuli are administered and identify, if any, patterns in their effects.

## Methods

A rapid review approach was used, also known as a rapid synthesis review (Hamel et al., 2021). This aimed to uncover the outcomes associated with olfactory stimulation in dementia care within a fixed timeframe and to systematically and transparently assess the effectiveness of olfactory interventions.

The protocol has been registered in the International Prospective Register of Systematic Reviews (PROSPERO) database (D’Andrea et al., 2020).

### Eligibility criteria

This review aimed to evaluate the use of olfactory stimuli in dementia care.

No geographical or time limits on the publication were imposed on the search. Studies were included based on the inclusion and exclusion criteria (for details, see Supplementary Material, Table S1).

### Search strategy

Guided by a specialist health librarian, the interdisciplinary review searched and identified all relevant published studies using the following databases: CINAHL, PsycINFO, Medline, PsycARTICLES, Academic Search Elite and Chemical Senses.

A combination of Boolean operators and truncations were used (see Supplementary Material, Table S2). Hand searching for references in included papers was conducted.

### Screening and selection

Electronic search results were downloaded into Rayyan software for semi-automated screening (Ouzzani et al., 2016). Two independent reviewers were involved in the screening and study selection (Tricco et al., 2017). A lead reviewer independently screened all articles by reading titles and abstracts against the inclusion and exclusion criteria. A random subset (20%) of electronic search results was independently screened by a second reviewer to minimise the risk of selection bias through inappropriate exclusion of relevant studies. Any discrepancies between the reviewers were resolved through discussion. Titles for which an abstract was not available or unclear were included for subsequent review of the full article. Where articles were not obtained through institutional holdings available to the reviewers, attempts were made to contact the author to procure the article. The lead reviewer assessed the study eligibility by reading their full text. In addition, backward citation searching and forward citation tracking were conducted on included articles to identify any missing studies. Any articles from the hand search that met the inclusion criteria were included for review.

### Quality assessment of studies

The Mixed Methods Appraisal Tool (MMAT) (Hong et al., 2018) was used by the lead reviewer to assess the methodological quality of the included studies. The second reviewer independently assessed a subset (20%) of articles. For each study, it has been provided a description of MMAT domains that were not addressed and how confident the authors were regarding the study findings based on the risk of bias assessed.

An overall quality score for each paper was presented using stars (*) that provide information on the risk of bias where 5-star indicates low risk, 4-star or 3-star moderate risk and 2-star or 1-star high risk. It is important to note that overall scores are arbitrary but useful for reporting the quality of included studies.

All eligible studies were included, and none was excluded based on quality assessment.

### Data extraction

This study used a single-reviewer extraction approach with a second reviewer checking the accuracy of extractions. For each included article, the lead reviewer extracted data using an Excel spreadsheet. The piloted extraction table included for each study: author/s, year of publication, country, design, participant information (i.e. sample size; age; subtype and stage of dementia), setting, aim, description of the intervention and stimuli, outcome measures and a summary of findings. The second reviewer checked for accuracy and completeness of the extracted data.

Study authors were not contacted for clarification or obtaining information in case of missing data.

### Data synthesis

A narrative synthesis was used. Included studies were grouped in relation to the domains investigated (e.g. responsive behaviour), scent type and olfactory delivery methods. A meta-analysis was not conducted due to the heterogeneity in the designs, interventions, outcomes and measurement tools used, as well as intervention effects.

To enhance transparency and replicability of the review, a Preferred Reporting Items for Systematic Reviews and Meta-Analyses (PRISMA) statement (Moher et al., 2009) was integrated with a list of key reporting items for rapid review (Tricco et al., 2017). This served as a guide to strengthen methodology and knowledge synthesis tailored to the objectives of the rapid review (for details see Supplementary Material, Table S3).

## Results

**Figure 1.**
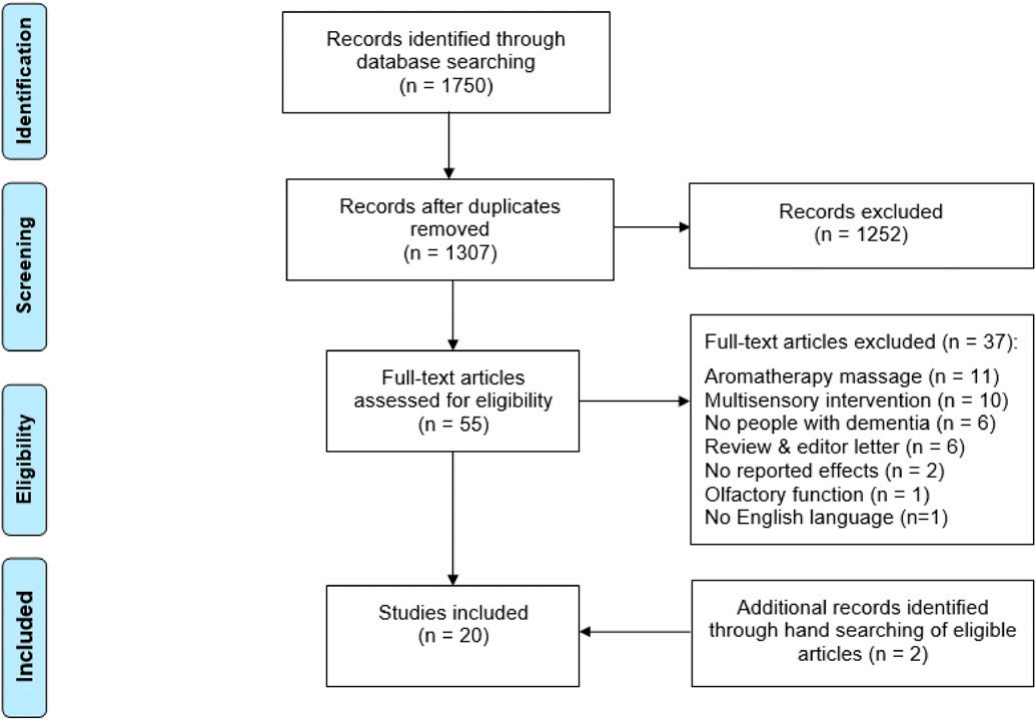
PRISMA flow diagram of study selection process.

Database searches up to and including 07 April 2021 returned 1307 articles after removing duplicates. A total of 55 articles were selected for full-text assessment and 20 (including two additions following reference check and forward citation tracking) were included in the review. An overview of the study selection process is shown in Figure 1.

### Study characteristics

Fourteen studies used a quasi-experimental design (El Haj et al., 2018; Glachet et al., 2019; Glachet & El Haj, 2019, 2020a , 2020b; Gray & Clair, 2002; Henry et al., 1994; Holmes et al., 2002; Jimbo et al., 2009; Lopis et al., 2021; Moorman Li et al., 2017; Snow et al., 2004; Sulmont-Rossé et al., 2018; Takeda et al., 2017), five studies used an experimental design (Fu et al., 2013; Lin et al., 2007; Sakamoto et al., 2012; Smallwood et al. 2001; Takahashi et al., 2020) and one was a case study (Brooker et al., 1997). No qualitative or mixed methods studies were identified from the search strategy used. An overview of the studies is given in Table 1.

**Table 1.** Overview of study characteristics.

References and country	Study design	Participants		Setting	Study aims	Intervention procedure	Measures
		N	Mean/range age				
Lopis et al., (2021), F	Quasi-experimental (non-randomised design; between-subject design)	180 60 PwD^r^ 60 HoP^n^ 60 young adult Mild AD^a^	80.9 ± 6.2 (exp) control: 80.1 ± 6.2 HoP^n^ 22.2 ± 2.9 young adult	-	Assess the frequency and phenomenological characteristics (emotional valence, emotional intensity, memory vividness and rarity) of odour-evoked autobiographic memories compared those recalled by visual and auditory cues	Participants were asked to recall memories after presenting and labelling either 4 odours, 4 sounds (i.e. cutting bread, crunching apple, wood crackling and wine bottle opening), or 4 pictures (French bread, apple, a wood fire, wine bottle)	MMSE^o^; Frontal Assessment Battery; verbal fluency task; episodic memory with 5-word test; forward and backward digit spans; 15-item Geriatric Depression Scale; The Mini International Psychiatric Interview 5.0.0; Likert scales for assessing emotional valence, emotional intensity, vividness and rarity of the memory as well as stimuli property (intensity; stimulus-reality; pleasantness)
Glachet & El Haj (2020a), F	Quasi-experimental (non-randomised design)	49 24 PwD^r^ (exp.) 25 HoP^n^ (control) Mild AD^a^	85.12 ± 5.68 (exp) 84 ± 8.5 (control)	-	Investigate the phenomenological characteristics (specificity, arousal and emotional valence) and retrieval time of past events and future thinking	Participants retrieved 1 past and 1 future event for 2 minutes in a free-odour condition and after odour exposure. The sessions were counterbalanced and 1 week apart	MMSE^o^; Grober and Buschke’s task; Geriatric Depression Scale; SAM^s^; TEMPau^v^; reaction time between the end of the instruction and the beginning of the narrative
Glachet & El Haj (2020b), F	Quasi-experimental (non-randomised design)	49 24 PwD^r^ (exp.) 25 HoP^n^ (control) Mild AD^a^	85.12 ± 5.68 (exp) 84 ± 8.5 (control)	-	Investigate the effects of odour exposure on access to self-concept (i.e. psychological self, physical self or social self)	Participants produced self-related statements of their identity (including roles, personality traits or physical traits) within 1 min following odour and odour-free exposure. The sessions were counterbalanced and 1 week apart	MMSE^o^; Grober and Buschke’s task; Geriatric Depression Scale; span task (forward and backward span) SAM^s^; phonemic and semantic fluency tasks; total number of self-related statements (i.e. psychological self, social self and physical self-statements)
Takahashiet al. (2020), JAP	Experimental (single-blind randomised controlled)	36 Mild-moderate AD^a^ Care staff	76.2 ± 9.8 (exp) 75.8 ± 7.8 (control)	-	Evaluate the effects of ethanol with and without aroma extracts on caregiver burden, residents' responsive behaviours and cognitive function	Exp. group was exposed to ethanol with cedar scent diffused in the living room and bedroom and sprayed into clothing and bedding a few times a day over an 8-week period. The control was exposed to ethanol without cedar scent. Effects were tested pre-intervention, after 4 weeks and post-intervention	OSIT-J^q^; NPI^p^; ZBI-J^x^; ADAS-cog^b^
Glachet et al. (2019), F	Quasi-experimental (non-randomised design; within-subject design)	54 26 PwD^r^ (exp.) 28 HoP^n^ (control) Mild AD^a^	72.69 (exp) 70.82 (control)	-	Assess the effect of odour exposure on the retrieval of recent and remote memories	Participants recalled two autobiographical memories related to childhood, adulthood and last 5 years in 1 free-odour and 1 odour (i.e. coffee) session	MMSE^o^; Grober and Buschke’s task; span tasks; HAD^l^; TEMPau^v^
Glachet & El Haj (2019), F	Quasi-experimental (non-randomised design)	48 25 PwD^r^ (exp.) 23 HoP^n^ (control) Mild AD^a^	82.04 ± 7.34 (exp); 80.91 ± 9.87 (control)	-	Explore the impact of olfactory cueing on autobiographic memories (i.e. arousal, emotional valence, subjective reliving and specificity) and its relationship with depression	Participants recalled autobiographical memories in 1 free-odour and 1 odour session	MMSE^o^; HAD^l^; SAM^s^; Grober and Buschke’s task; subject reliving; TEMPau^v^
El Haj et al. (2018), F	Quasi-experimental (non-randomised design)	58 28 PwD^r^ (exp.) 30 HoP^n^ (control) Mild AD^a^	73.25 ± 6.71 (exp) 71.75 ± 8.05 (control)	-	Compare the specificity, emotion, retrieval time of odour-, music-evoked and no sensory cueing autobiographical memories	Participants recounted 2 personal events for 3 minutes after 1 odour exposure, 1 music exposure and 1 control session. Between the two retrievals, an executive task was performed (i.e. odour-verbal fluency; music- plus–minus task; control condition-Stroop task). The sessions were counterbalanced with a 3- to 5-day interval between sessions	MMSE^o^; Grober and Buschke’s task; HAD^l^; executive function: Verbal fluency task, the plus–minus task, Stroop task; TEMPaus^v^; emotion and mental time travel rate Likert-scale; reaction time between the end of the instruction and the start of memory generation
Sulmont-Rossé et al. (2018), F	Quasi-experimental (non-randomised crossover design)	32 Moderate to severe AD^a^	86.8 mean 75-98 range	Nursing home	Evaluate the impact of olfactory priming in food intake and eating behaviours	Participants took part in a total of (alternated) 2 control lunches and 2 primed lunches, every 2 weeks. Room odourisation started 15 min before lunch and ended before serving the main course	MMSE^o^; food intake: Weighing plates before and after consumption; proxy observation of resident’s behaviours during lunch time (attention to care staff, to dish and meal tray before lunch; appetite; sit at the table)
Moorman Li et al. (2017), US	Quasi-experimental (pre- and post-design)	23	83 mean 73–97 range	Adult day care centre	Evaluate the effects of aromatherapy on responsive behaviours (i.e. restlessness/wandering, agitation, anger and anxiety) and comparison of age cohorts, gender and individual behaviour frequency	Lavender oil was diffused in a room for 20 min twice a day (morning and mid-afternoon) for 2 months. Pre- (a 2-months) and post-intervention observation were conducted	Behaviour/intervention monthly flow record observation pre- and post-intervention
Takeda et al. (2017), JAP	Quasi-experimental (non-randomised crossover design)	19 Severe dementia	80.7 ± 9.1	Nursing home	Evaluate the effects of aromatherapy on symptoms of sleep disturbance	The residents’ pillows were wrapped for 20 days with a towel with no odour, followed by 20 days with a scented towel during the night	MMSE^o^; FIM^i^; times of going to bed and rising; NPI^p^; 24 h sheet-type body vibrometer; sleep disturbance: (1) difficulty initiating sleep; (2) difficulty maintaining sleep; (3) early morning awakening and (4) daytime disorder
Fu et al. (2013), AU	Experimental (single-blind randomised controlled)	67 9 mild 23 moderate 29 severe 29 dementia 16 AD^a^ 3 VaD^w^ 8 cognitive impairment (no diagnosis) 5 other dementia	84 ± 6.36 61–93 range	Long-term care facilities	Compare the effect of aromatherapy (oil spray), placebo (water spray) and a combination of aromatherapy and hand massage to reduce responsive behaviours, particularly aggression and agitation	3 groups received a combination of aromatherapy and hand massage, or aromatherapy (lavender spray on the upper chest), or placebo control (water spray on the upper chest), twice a day (9–11 a.m.; 2–4 p.m.) 7 days a week for 6 weeks. Hand massage duration: 5 min (2.5 min for each hand). Evaluation occurred at 5 points time (baseline, week 2-4–6, post-test)	MMSE^o^; CMAI-SF^d^
Sakamatoet al. (2012), JAP	Experimental (double-blind randomised controlled)	145 72 (exp) 73 (control) Moderate dementia	84.2 ± 7.8 (exp) 84.1 ± 7.7 (control)	Nursing homes	Investigate the effects of lavender on fall incidence in nursing home residents	24 h olfactory stimulation from a lavender patch attached to the inside of the resident’s clothes near the neck for 360 days	Number of resident falls; Barthel Index; Vital Index; St. Thomas’s Risk Assessment Tool in Falling Elderly Inpatients; MMSE^o^; CMAI^c^
Jimbo et al. (2009), JAP	Quasi-experimental (non-randomised crossover design)	28 PwD^r^ 21 care staff 9 mild to moderate 19 Severe 17 AD^a^ 3 VaD^w^ 8 other dementia	86.1 ± 6.9	-	Assess the effect of aromatherapy on cognitive function	Participant received aromatherapy in two different rooms in the morning and evening for 28 days. The intervention was preceded by a control period of 28 days and followed by a 28-days wash out period. During the control and wash up period, participants did not receive any intervention. Assessment at four points: Before control condition, before and after aromatherapy and after a wash out period	GBSS-J^k^; TDAS^u^ FAST^h^; HDS-R^m^; head computed tomography; blood analysis and biochemical examination; ZBI-J^x^
Lin et al. (2007), JAP	Experimental (crossover randomised design)	70 Moderate to severe 44 AD^a^ 21 VaD^w^ 5 other dementia	78.29 ± 4.06 69–89 range	Care homes	Compare the effect of lavender aromatherapy with a control condition (sunflower odour)	Each participant received both conditions. Both conditions lasted for 3 weeks and were 2 weeks apart. At least 1 h of exposure to odour during sleep time at night. Evaluation occurred pre- (0 week), post-intervention (week 3) and pre- (week 5), post-control condition (week 8)	CMAI^c^ Chinese version; CNPI^f^ CMMSE^e^
Snow et al. (2004), US	Quasi experimental (non-randomised crossover design)	7 Severe dementia AD^a^	-	Nursing home	Investigate effects of an essential oil on the frequency of agitated behaviours; participants’ olfactory functions	Each participant had an absorbent fabric sachet with aroma [lavender (A) and thyme (B)] and no aroma oil [unscented grapeseed C)] pinned to their shirt near the collarbone every 3-h, for a total of 3 applications per day, over 2 weeks for each condition (total of 10 weeks). The condition followed ABCBA order	CMAI^c^; SIRS^t^; olfactory functioning: Identification (sniff and name task), discrimination (2-odours discrimination task); participants’ reactions (recorded verbatim in the above two tasks)
Holmes et al. (2002), UK	Quasi-experimental (non-randomised crossover design)	15 Severe dementia 4 AD^a^ 7 VaD^w^ 3 DLB^g^ 1 FTD^j^	79 ± 6.3	Long-term care facility	Evaluate the effect of aromatherapy steam on agitated behaviour	Each participant was exposed for 2h (4–6 p.m.) in a communal area to a total of 5 odour and 5 placebo (water) sessions on alternate days, over a period of 2 weeks	Pittsburgh Agitation Scale
Gray & Clair (2002), US	Quasi-experimental (non-randomised crossover design)	13	-	Care homes	Examine the effects of aromatherapy on the administration of medications (i.e. frequencies of resistive behaviours, time of administer medications, gender difference for frequency and time-administration)	Twenty minutes before early morning medication administration, a cotton ball with essential oil (lavender vera, sweet orange or tea tree) or without aroma (control) was taped to the lapel of each participant. Each of the four conditions was repeated in a random order four times for a total of 16 administrations	Video records for the duration of the medication administration
Smallwood et al. (2001), UK	Experimental design (single-blind randomised control design)	21 Severe dementia	66.8 ± 11.5	Hospital	Compare the impact of aromatherapy massage, plain oil massage and aromatherapy and conversation on responsive behaviours	Participants were randomly allocated in aromatherapy massage, or massage or aromatherapy intervention provided twice weekly	15 minutes video records in 4 times a day (10–11 a.m., 11–12 noon, 2–3 p.m. and 3–4 p.m.) twice during 2-weeks at the baseline. Participants’ behaviours were recorded after receiving intervention
Brooker et al. (1997), UK	Single case study	4 Severe dementia 2 AD^a^ 1 AD^a^ and Parkinson 1 FTD^j^	74–91 range	Hospital	Evaluate the impact of aromatherapy, aromatherapy massage, massage only and no treatment on agitation	Each participant randomly received between 8 and 12 sessions of each four conditions (lavender oil delivered via fan; lavender and massage; massage; no treatment) over a 3-month period. Each session lasted 30 minutes	Agitation observation scale 1 h after intervention, rated at 1 minute intervals
Henry et al. (1994), UK	Quasi experimental (pre- and post-design)	9 Severe dementia -	-	Hospital	Assess the effect of aromatherapy on the number of night time hours spent asleep	Each participant was exposed to lavender aromatherapy in the bedroom during the night. Over a 7-week period: 2 weeks sleep observation; the third week the lavender was diffused only in the female dormitory; the fourth week only in the male dormitory; the final 3 weeks in both dormitories	Tot. hour sleep: Sleep observation between 12 a.m. to 7.30 a.m. at half hourly intervals

^a^AD = Alzheimer’s Disease.

^b^ADAS-cog = Alzheimer’s Disease Assessment Scale-Cognitive Subscale.

^c^CMAI = Cohen-Mansfield Agitation Inventory.

^d^CMAI-SF = Cohen-Mansfield Agitation Inventory Short Form.

^e^CMMSE = Mini-Mental State Examination Chinese version.

^f^CNPI = Neuropsychiatric Inventory Chinese version.

^g^DLB = Dementia Lewy Body.

^h^FAST = Functional Assessment Staging Test.

^i^FIM = Functional Independence Measure.

^j^FTD = Frontotemporal dementia.

^k^GBSS-J = The Gottfries, Bråne, Steen Scale.

^l^HAD = Hospital Anxiety and Depression Scale.

^m^HDS-R = Hasegawa’s dementia scale.

^n^HoP = Healthy older People.

^o^MMSE = Mini-Mental State Examination.

^p^NPI = Neuropsychiatric Inventor.

^q^OSIT-J = Odor Stick Identification Test for Japanese.

^r^PwD = People with Dementia.

^s^SAM = Self-Assessment Manikin.

^t^SIRS = Severe Impairment Rating Scale.

^u^TDAS = Touch-panel type Dementia Assessment Scale.

^v^TEMPau = Test Episodic de Mémoire du Passé.

^w^VaD = Vascular dementia.

^x^ZBI-J = Zarit Caregiver Burden Interview Japanese version.

The included studies varied greatly in terms of stages and subtypes of dementia. Six studies included people with mild dementia (El Haj et al., 2018; Glachet et al., 2019; Glachet & El Haj, 2019, 2020a, 2020b; Lopis et al., 2021), one with moderate dementia (Sakamoto et al., 2012) and six with severe symptoms (Brooker et al., 1997; Henry et al., 1994; Holmes et al., 2002; Snow et al., 2004; Smallwood et al., 2001; Takeda et al., 2017). Five other studies included those with different stages of dementia, including mild to moderate and moderate to severe (Fu et al., 2013; Jimbo et al., 2009; Lin et al., 2007; Sulmont-Rossé et al., 2018; Takahashi et al., 2020). Two studies did not report participants’ stage of dementia (Gray & Clair, 2002; Moorman Li et al., 2017). All studies providing information about the subtype of dementia included people with Alzheimer’s disease (AD). Among them, three studies (Fu et al., 2013; Jimbo et al., 2009; Lin et al., 2007) included people with vascular dementia (VaD), frontotemporal dementia (FTD) and ‘other dementias’; Brooker et al.’s sample (1997) included AD and FTD; whereas Holmes et al. (2002) included participants with AD, VaD, FTD and dementia Lewy body (DLB). Subtypes of dementia were not provided in six studies (Gray & Clair, 2002; Henry et al., 1994; Moorman Li et al., 2017; Sakamoto et al., 2012; Smallwood et al., 2001; Takeda et al., 2017).

Among the 20 studies, eight used a control group composed of older people of similar age to those with dementia (*n* = 5) (El Haj et al., 2018; Glachet et al., 2019; Glachet & El Haj, 2019, 2020a, 2020b), people with dementia with the same demographic characteristics to the experimental group (*n* = 2) (Sakamoto et al., 2012; Takahashi et al., 2020) and older and young people (*n* = 1) (Lopis et al., 2021). Jimbo et al. (2009) and Takahashi et al. (2020) included care staff and caregivers in their sample.

Only eight studies (Brooker et al., 1997; Glachet & El Haj, 2020a, 2020b; Gray & Clair, 2002; Lopis et al., 2021; Snow et al., 2004; Sulmont-Rossé et al., 2018; Takahashi et al., 2020) conducted olfactory screening of participants pre-intervention using standardised tools such as Odor Stick Identification Test-Japanese version (Saito et al., 2006), a Likert self-assessment scale (e.g. Pouliot & Jones-Gotman, 2008) or by recording the participants’ verbal and non-verbal reactions to odorants (e.g. coffee).

### Olfactory stimuli

Across the 20 different studies, a total of 20 different olfactory materials were used.

The smells used were mainly pure, diluted or in a mixture of two or more scents.

Only a few studies reported concentration and dosage information (Fu et al., 2013; Holmes et al., 2002; Jimbo et al., 2009; Lin et al., 2007; Snow et al., 2004; Takahashi et al., 2020) (see Supplementary Material, Table S4, for a summary of the scents used, study domains and administration methods).

The selection of olfactory materials was based on physical and physiological effects as reported by previous studies or participants’ odour preferences assessed pre-intervention (in three studies): Takeda et al. (2017) asked participants to select and express their preference for one of three oils presented, whereas Glachet and El Haj (2020a, 2020b) used olfactory items that were rated by participants as easy to detect and familiar. One study did not record the rationale for the smells chosen.

Lavender was the most commonly used scent. This essential oil was used in 13 studies primarily to reduce responsive behaviours (*n* = 8) (Brooker et al., 1997; Gray & Clair, 2002; Holmes et al., 2002; Fu et al., 2013; Li et al., 2007; Moorman Li et al., 2017; Smallwood et al., 2001; Snow et al., 2004) such as agitation, falls (*n* = 1) (Sakamoto et al., 2012), to improve sleep patterns (*n* = 2) (Henry et al., 1994; Takeda et al., 2017) or cognitive function (*n* = 1) (Jimbo et al., 2009). Among these studies, two articles (Jimbo et al., 2009; Takeda et al., 2017) combined lavender oil with sweet orange oil for their calming properties.

Orange and coffee were the next most common scents used. Coffee was used to explore and evaluate participants’ autobiographic memories in five French studies (El Haj et al., 2018; Glachet et al., 2019; Glachet & El Haj, 2020a, 2020b, Lopis et al., 2021), suggesting that this is a distinctive smell which is likely to be associated with an individual’s past. Orange was used for a variety of reasons, including eliciting memories (Glachet & El Haj, 2020a, 2020b), reducing responsive behaviours (Gray & Clair, 2002), increasing sleep (Takeda et al., 2017) and enhancing cognitive function (Jimbo et al., 2009).

### Olfactory administration methods

Olfactory stimuli were administered using a variety of methods and procedures.

### Inhalation

An inhalation method was used in 15 studies (Brooker et al., 1997; El Haj et al., 2018; Glachet et al., 2019; Glachet & El Haj, 2019, 2020a, 2020b; Henry et al., 1994; Holmes et al., 2002; Jimbo et al., 2009; Lin et al., 2007; Lopis et al., 2021; Moorman Li et al., 2017; Smallwood et al. 2001; Sulmont-Rossé et al., 2018; Takahashi et al., 2020). Among these, eight used diffusers, such as fans or steam diffusers (Brooker et al., 1997; Henry et al., 1994; Holmes et al., 2002; Jimbo et al., 2009; Lin et al., 2007; Moorman Li et al., 2017; Smallwood et al. 2001; Sulmont-Rossé et al., 2018). Lin et al. (2007) placed two diffusers next to the participants’ pillows for at least 1 hour during sleep at night. Similarly, Henry et al. (1994) diffused lavender oil in the participants’ bedrooms overnight using an electric fan. In another study, participants were exposed to a mixture of essential oils with stimulating properties (lemon and rosemary oil) for 2 hours in the morning and with calming properties (lavender and orange oils) for 90 min in the evening (Jimbo et al., 2009). Two other studies administered lavender oil twice a day. Moorman Li et al. (2017) diffused lavender for 20 min in a common area of a day care centre in the morning and in the mid-afternoon. Smallwood et al. (2001) diffused the lavender oil in a room twice a week across four times during the day (before 10 a.m., 11 a.m., 2 p.m., and 3 p.m.) for a total of eight sessions over 4 weeks.

Lavender oil was diffused in participants’ bedrooms for 30 min in 8–12 sessions over a 3-month period (Brooker et al., 1997) and in communal area for 2 hours in 10 sessions over 1 month (Holmes et al., 2002). Meat aroma (‘sauté de boeuf’, lit. ‘beef stir-fry’) was diffused in a nursing home’s dining room 15 min before lunch as olfactory priming to trigger food-related mental representations, aiming to stimulate appetite (Sulmont-Rossé et al., 2018).

Two studies used diffusers and sniffing sticks (Lopis et al., 2021; Takahashi et al., 2020) and five studies used scent bottles (El Haj et al., 2018; Glachet et al., 2019; Glachet & El Haj, 2019, 2020a, 2020b). Participants were asked to place the bottles under their nose and breathe normally, whilst closing their eyes and mouth. This procedure was conducted when participants were asked to retrieve memories, self-related statements or think about future events.

### Fabric and patch

Sakamoto et al. (2012) used a scent-infused lavender paper patch attached to the inside of the resident’s clothes near the neck for 24 h for 360 days. A similar method of administration was used by Gray and Clair (2002). A scented cotton-ball was taped to the lapel of each resident 20 min before the morning medications for 4 days for each of the three scents used. Snow et al. (2004) applied lavender oil for 2 weeks and thyme oil for the following 2 weeks to an absorbent fabric sachet. Takeda et al. (2017) applied essential oil to a towel wrapped around participants’ pillows for 20 days.

### Spray

Sprays were used in two studies. In one, lavender or water (control group) was sprayed directly onto individuals’ skin on their upper chest (Fu et al., 2013). In the other study, the aroma was sprayed on participants’ clothing and bedding a few times a day (Takahashi et al., 2020).

### Intervention effects

The effects of olfactory stimulation in each of the domains investigated in the studies included in the review are discussed in detail below and summarised in Table 2.

**Table 2.** Summary of the research outcomes and quality assessments.

References	Findings	MMAT score
Lopis et al. (2021)	A higher number of memories were recalled by older people, followed by PwD^f^ and young adult. Visual stimuli evoked significantly more (*p* < .05) and rarer (*p* < .05) memories than odours, and odours stimuli produced more memories than auditory stimuli in PwD^f^. No significant differences were found in emotional valence and vividness memories between groups and type of sensory stimuli. PwD^f^ (*p* = .01) and older people (*p* < .05) rated their memories significantly more emotional intense than the young adult group; no difference was found for the type of stimuli. PwD^f^ evoked significantly more memories in the age between 0–18 (*p* < .05); no differences for type of stimuli and age were found	***
Glachet and El Haj (2020a)	Significant increase in both groups in phenomenological characteristics of past and future (apart specificity for control group) events after odour-exposure. Significantly shorter reaction time (*p* = .001) for past event in PwD^f^ after odour exposure; significantly shorter reaction time for the control group for past event (with or without odour, respectively *p* = .01 and *p* = .005) and future event (with odour) (*p* = .03)	***
Glachet and El Haj (2020b)	Significant increase of the number of self-related statements in odour condition compared to odour-free condition in PwD^f^ (*p* < .001) and control group (*p* < .05). Significant increase of psychological self-statements in odour condition for PwD^f^ (*p* < .05). No difference in social and physical statements in both condition and groups	***
Takahashi et al. (2020)	Significant decreases (*p* < .05) in agitation, anxiety and irritability in the exp. group at 4 and 8 weeks. No significant difference in cognitive function between the two groups. Significantly lower caregiver burden (*p* < .05)	*
Glachet et al. (2019)	Significant increase in number of childhood (*p* < .05), adulthood (*p* < .01) and recent (*p* < .01) memories after odour-exposure than without odour. PwD^f^ significantly retrieved more specificity childhood (*p* < .01), adulthood (*p* < .01) and recent (*p* < .01) memories after odour exposure compared to odour-free condition. Regarding the temporal gradient of memories, PwD^f^ produced more adulthood memories than childhood memories and more childhood memories than recent memories with or without odour exposure	***
Glachet and El Haj (2019)	Significantly higher arousal (*p* < .01), subjective reliving (*p* < .05), specificity (*p* < .01) and positive (*p* < .01) odour-evoked autobiographical memories than for memories evoked without odour only in PwD^f^. Negative correlation between depression scores and emotional valence, arousal and subjective reliving in PwD^f^	***
El Haj et al. (2018)	Memories retrieved after odour and music exposure in PwD^f^ had higher specificity, emotional arousal, mental time travel and shorter retrieval time than in the control condition. Retrieval time was much shorter after odour exposure than music exposure	****
Sulmont-Rossé et al. (2018)	A significant effect of olfactory priming in meat food intake (*p* = .04). A positive effect in vegetable consumption (*p* = .06) compared to the control condition. Significant increase in resident’s interest toward the meal in the primed lunch. This effect was no longer observed when the priming session was replicated 2 weeks later with the same priming odour and menu	****
Moorman Li et al. (2017)	Non-significant reduction (*p* = .06) in the frequency of responsive behaviours pre- and post-intervention. In the analysis of individual responsive behaviours, significant decrease only for the frequency of agitation pre- and post-intervention. Participants in the 70–85 age cohort showed a significant decrease in agitation than the 86–100 cohort post-intervention. There was no significant difference for effects of gender on any of the four behaviour responses investigated	**
Takeda et al. (2017)	Total sleep time (*p* < .05) and sustained sleep period (*p* < .05) were significantly longer in the intervention period than in the control. Early morning waking in the intervention period was significantly less (*p* = .01) compared to that in the control. Total daytime sleep could not be adequately measured, and it was omitted from the analysis. No significant differences in other sleep measurements were observed	****
Fu et al. (2013)	No significant effect was found following aromatherapy alone and aromatherapy combined with massage on participants’ responsive behaviours	****
Sakamato et al. (2012)	Fewer falls in the lavender group, significant decrease in CMAI^c^ (*p* = .04) from baseline to 12-month follow-up. No difference between the two groups for any of the outcomes investigated	***
Jimbo et al. (2009)	A significant improvement in four GBSS-J^e^ items (*p* < .05) and TDAS^g^ (*p* < .05) after aromatherapy. Participants with AD^a^ showed significant improvement in total TDAS^g^ scores (*p* < .01). Blood analysis and biochemical examination showed no significant changes. Results from ZBI-J^h^ score showed no significant changes	***
Lin et al. (2007)	Significant effects were found in CCMAI^b^ (*p* < .001) and CNPI^d^ (*p* < .001) after odour condition. Independent sub-analysis showed no significant difference on odour condition response based on gender and subtype of dementia	***
Snow et al. (2004)	No significant treatment effects were found following the two odour conditions compared the control condition	**
Holmes et al. (2002)	Nine residents (60%) showed an improvement, five (33%) showed no change and one participant (7%) showed a worsening of agitated behaviour during aromatherapy compared with placebo	***
Gray and Clair (2002)	No significant difference in behaviours or duration of medication administration and gender influence across the four conditions	**
Smallwood et al. (2001)	No significant difference between the treatments, although consistent reduction in agitation following the aromatherapy massage. Significant time difference occurred between 3 and 4 p.m. between aromatherapy massage (*p* < .05) and only aromatherapy (*p* = .05)	***
Brooker et al. (1997)	Findings varied considerably between individuals. The observations showed benefit for two people only following just aromatherapy or massage. Other two participants reported an increase of agitation following all treatment conditions apart the aromatherapy-massage for one of them	***
Henry et al. (1994)	A significant increase in the total of hours slept following aromatherapy (*p* < .01)	*

^a^AD = Alzheimer’s disease.

^b^CCMAI = Cohen-Mansfield Agitation Inventory Chinese version.

^c^CMAI = Cohen-Mansfield Agitation Inventory.

^d^CNPI = Neuropsychiatric Inventory Chinese version.

^e^GBSS-J = The Gottfries, Bråne, Steen Scale.

^f^PwD = People with dementia.

^g^TDAS = Touch-panel type Dementia Assessment Scale.

^h^ZBI-J = Zarit Caregiver Burden Interview Japanese version. Risk of bias: (*****) low; (****) or (***) moderate; (**) or (*)high.

### Responsive behaviours

Mixed findings on the effect of olfactory stimulation on responsive behaviours were reported.

Moorman Li et al. (2017) reported a significant decrease in the frequency of observed agitation following 2 months of a scent exposure during activities in a day care centre. These improvements were not found in other domains observed (restlessness/wandering, anger and anxiety). The decrease in agitation was significantly larger in participants aged 70–85 age compared to those aged 86–100. There was no gender difference in the results in all four domains.

Improvements in responsive behaviours such as agitation, anxiety and irritability were also reported by Takahashi et al. (2020) in the experimental group after environmental exposure to an ethanol cleaning solution with added cedar fragrance and distilled solution with cedar sprayed on clothing and bedding, compared to the control group who were exposed to the ethanol solution without fragrance.

Sakamoto et al. (2012) found a significant decrease in the Cohen-Mansfield Agitation Inventory (CMAI) (Cohen-Mansfield & Kerin, 1986) score following a 12-month period of olfactory stimulation using a patch worn by residents in nursing homes. A significant decrease was also found in the Neuropsychiatric Inventory (NPI) Chinese version (Leung et al., 2001) and CMAI scores after a 3-week period of 1 hour of lavender exposure at night compared to the control condition, that is, the same procedure with sunflower oil (Lin et al., 2007). Sub-analysis showed no significant difference in odour condition response based on gender and subtype of dementia (i.e. AD and VaD).

Four studies did not report significant benefits after olfactory stimulation in people with dementia (Fu et al., 2013; Gray & Clair, 2002; SmalIwood et al., 2001; Snow et al., 2004). SmalIwood et al. (2001) administered lavender oil or a control oil either via a diffuser or massage, twice a week for 4 weeks. Analysis of video recordings of participants’ motor behaviours after two aromatherapy treatments (diffuser or massage) and the placebo conditions found no significant differences between the three groups. Snow et al. (2004) assessed the effect of an infused fabric sachet attached to clothing for two different aromas and one with no aroma for a total of 10 weeks. No statistical difference on the CMAI scale was reported across the three conditions. No significant effects were found in Fu et al.’s (2013) study. This used olfactory stimuli via oil spray on residents’ upper chests and compared this to aromatherapy hand massage and placebo (water spray) for 6 weeks. Gray and Clair (2002) examined the effects of an infused cotton-ball taped to the lapel of resident for 20 min, whilst medications were administered in terms of frequencies of resistive behaviours, time taken to administer medications and gender difference. No significant differences occurred in behaviours, duration of medication administration and gender across four conditions: a cotton-ball without odour and with lavender, sweet orange or tea tree.

Mixed findings were reported in two studies (Brooker et al., 1997; Holmes et al., 2002). Brooker et al. (1997) reported that two participants had reduced agitation following the aromatherapy intervention, whereas two other participants showed increased agitation. Similarly, Holmes et al. (2002) found that nine residents showed an improvement, five reported no change and one participant had increased agitation following an aromatherapy intervention compared with a placebo. Taking into account the subtypes of dementia, three participants with AD showed positive benefit, one reported no change. Of the seven participants with VaD, five showed improvement and two showed no change. Of the three people with a diagnosis of DLB two showed no change, one person worsened and the only participant with FTD showed reduced agitation.

### Autobiographical memory

Five studies reported that smell is an effective cue for triggering autobiographic memories with one suggesting that it can facilitate future thinking (the capacity to project oneself into the future) (El Haj et al., 2018; Glachet et al., 2019; Glachet & El Haj, 2019, 2020a, Lopis et al., 2021). All studies compared the participants’ responses following one session of odour exposure and one session with no odour (control condition), apart from Lopis et al. (2021) who conducted a session using pictures as comparison to the olfactory stimuli and El Haj et al. (2018) who conducted three sessions: odour exposure, music exposure, and control condition.

Glachet and El Haj (2019) found that odour-evoked memories were more positive, specific, emotional and evocative compared to memories triggered in the odour-free condition. Additionally, El Haj et al. (2018) found that odour-evoked autobiographical memories had a shorter retrieval time compared to memories triggered following music exposure. Similar findings were reported in a study by the same group (Glachet & El Haj, 2020a) that evaluated the effect of odour exposure on past events and future thinking. Participants exposed to the odour condition reported past and future events with higher phenomenological characteristics (i.e. specificity, arousal and emotional valence) and shorter retrieval time for past events but not for future events which was found only in the control group. Glachet et al. (2019) also reported that olfactory stimuli triggered a significantly higher number of recent (i.e. last five years) and remote (childhood and adulthood) memories compared to an odour-free condition. While, a more recent study (Lopis et al., 2021) comparing the impact of odour, auditory and visual cues in retrieval of autobiographic memories found that visual stimuli led to recall of more and rarer memories and overall, a better retrieval performance across auditory and odour stimuli. Furthermore, odour-evoked memories were not significantly more emotional than those recalled following pictures or sounds.

### Cognitive function

Mixed results were reported for the benefits of olfactory stimulation on cognitive functions in people with mild to moderate dementia, with one study showing positive effects (Jimbo et al., 2009) and one no effects (Takahashi et al., 2020).

Jimbo et al. (2009) investigated cognitive function after exposure to two scent mixtures with stimulating and calming properties. The results showed a significant improvement in the scores of four items of the Japanese version of the Gottfries, Bråne, Steen Scale (GBSS-J) (Homma et al., 1991) and the overall score of Touch-panel type Dementia Assessment Scale (TDAS) (Inoue et al., 2011). Interestingly, participants with a diagnosis of AD greatly improved in the TDAS (*p* < .01) compared to the other participants. In contrast, no significant difference in cognitive function was found in Takahashi et al.’s study (2020) between the control and experimental group.

### Self-concept

Glachet and El Haj (2020b) evaluated the role of smell as a cue to enhance the retrieval of self-related knowledge (i.e. self-concept). It includes the psychological, physical and social self-related mental representations about who we are (e.g. traits, beliefs, values, social status, roles and physical attributes) and includes cognitive and affective judgements about ourselves. The authors reported that participants exposed to the odour condition generated significantly more self-related statements in response to the question ‘Who am I?’ compared to the odour-free condition. In particular, smells triggered more self-statements associated with the psychological dimensions of the self.

### Sleep

Two studies supported the use of olfactory stimulation to reduce sleep disturbance in people with severe dementia (Henry et al., 1994; Takeda et al., 2017).

Henry et al. (1994) found a significant increase in total hours slept after 4 weeks of exposure to a room scent overnight compared to an odour-free condition. Takeda et al. (2017) reported a significant effect when using aromatherapy overnight, including longer total sleep duration, sustained sleep period and less early morning waking. Sleep patterns and residents’ behaviours were measured by comparing the data from the NPI (Cummings et al., 1994) and a 24-h body movement monitoring device collected during the 20 days when the resident’s pillow was wrapped in a towel without oil (control condition) and the following 20 days when the essential oil was introduced to the towel surface.

### Appetite

One study assessed the effect of olfactory stimulation on food intake (Sulmont-Rossé et al., 2018), in which participants were exposed to a meat scent in the dining room for 15 min before serving the main course during two lunches that were alternated with the control condition (two scent-free lunches). A significant effect of the olfactory priming was found with a 25% increase in meat consumption and an increase in vegetable consumption approaching significance compared to the control condition. Behavioural observations also showed a significant increase in residents’ interest in the meal in the scent-primed lunch condition. However, this effect was no longer observed when the intervention was replicated 2 weeks later with the same priming odour and the same menu.

### Balance

Positive results were found in the only study in this review focusing on the effect of smells on balance. Sakamoto et al. (2012) reported that nursing home residents who wore a lavender patch for almost a year experienced fewer falls and incident rates compared to those who did not wear a patch.

### Study quality

Key methodological issues in the RCT studies include poor quality and reporting of the randomisation process, participants’ adherence to the study, comparability of the experimental and control group at baseline and completeness of outcome data reported. In the majority of quantitative non-randomised studies, it was unclear whether the interventions were administered as intended and if confounders were considered in the design and analysis, due to a lack of information. The risk of bias identified in the single case study included in the review was lack of data presented in the paper and information on participants’ inclusion and exclusion criteria.

An overall quality score for each study was developed by rating each MMAT domain as 1 if the study reported appropriate information and as 0 (zero) if the domain was not met or if the information reported was unclear. The highest score was 4 and the lowest was 1. Five out of 20 studies had a MMAT score of 2-star or less. The scores are presented in Table 2. For each of the outcomes evaluated in this review, a brief summary of the studies bias and confidence in the results is reported below.

Two studies (Moorman Li et al., 2017; Takahashi et al., 2020) out of four demonstrating improved responsive behaviours were assessed at high risk of bias. This was due to a lack of strategies to reduce the effect of any potentially confounding factors (Moorman Li et al., 2017), a single un-blinded observation (Moorman Li et al., 2017; Takahashi et al., 2020) and a lack of information on study adherence (Moorman Li et al., 2017), randomisation and completeness of outcomes data (Takahashi et al., 2020). There is moderate confidence in the results reported by the other two studies on responsive behaviours included in the review due to the incomplete outcome data and the high number of participants withdrawing (137 out of 282) in Sakamoto et al.’s (2012) study, the lack of blinding of outcomes assessment and a lack of information regarding the intervention adherence in Lin et al.’s (2007) study. Among the six studies demonstrating no or mixed effects of olfactory stimulation on responsive behaviours, four studies were assessed at moderate risk of bias (Brooker et al., 1997; Fu et al., 2013; Holmes et al., 2002; SmalIwood et al., 2001) and two presented high risk due to the lack of information on the confounder analysis (Gray & Clair, 2002; Snow et al., 2004), study adherence (Snow et al., 2004), study deviation (Gray & Clair, 2002) and small sample size (*n* = 7) (Snow et al., 2004).

There is moderate confidence in the results of studies on autobiographic memories which were evaluated with a moderate risk of bias due to unclear information regarding adherence to the protocol (El Haj et al., 2018; Glachet et al., 2019; Glachet & El Haj, 2019; Lopis et al., 2021), numbers of participants withdrawing (Glachet & El Haj, 2019, 2020a; Lopis et al., 2021) and confounder analysis (Glachet et al., 2019; Glachet & El Haj, 2020a).

Confidence in Henry et al.’s (1994) study results regarding the impact of olfactory intervention on sleep was low due to a lack of information about the sample characteristics, study adherence and completeness of outcomes data. Takeda et al.’s (2017) study was evaluated with moderate risk of bias in all domains of the MMAT apart from the sample representative of the target population due to the small sample size (*n* = 19).

The studies evaluating the effect of olfactory stimuli on appetite (Sulmont-Rossé et al., 2018), falls (Sakamoto et al. 2012), cognitive function (Jimbo et al., 2009) and self-concept (Glachet and El Haj, 2020b) were assessed at moderate risk of bias: a lack of information on the study compliance (Glachet and El Haj, 2020b; Jimbo et al. 2009), confounder analysis (Jimbo et al. 2009; Sulmont-Rossé et al., 2018) and numbers of participants withdrawing (Glachet and El Haj, 2020b; Sakamoto et al. 2012). For a summary of risk of bias, see Supplementary Material, Figure S1.

## Discussion

Twenty studies were included in this review, exploring the effects of olfactory stimulation in relation to three domains: responsive behaviour, cognitive function, and physical functioning.

In line with previous reviews (e.g. Ball et al., 2020), the findings from the current review showed that olfactory interventions were not associated consistently with decreasing frequency of responsive behaviours for people with dementia exposed to olfactory stimulation. These findings arose from 10 studies included in the review assessing responsive behaviours, among which four reported positive outcomes, four found no significant effect of olfactory stimulation, and the effects observed in two studies reported variable responses.

The extent to which olfactory intervention could improve cognitive functioning is unclear due to mixed findings and limited evidence. The current review confirms olfactory stimuli as effective cues to stimulate positive, emotional, specific and less considered autobiographic memories in people with dementia. Glachet and El Haj (2020b) demonstrated that odour exposure can positively impact the individual conceptual self-related knowledge (self-concept). The findings of the present review suggest that olfactory stimuli could play a role in supporting the identity of people with dementia as they enhance autobiographic memories and access to self-concept.

A surprisingly limited body of evidence was found on the impact of olfactory stimulation on physical functioning, such as sleep, food intake and balance. This is despite literature stating that smell cues can modify eating behaviours (Zoon et al., 2016), enhance sleep pattern (Park et al., 2016) and improve postural control (Freeman et al., 2009). Although limited, the current review provides evidence of the benefit on the total and sustained hours slept of people with dementia following overnight odour exposure for 3–4 weeks. A significant odour priming effect was found for food intake. However, the increase in meat and vegetable consumption observed was not noted on the second exposure to the same odour priming after 2 weeks (Sulmont-Rossé et al., 2018). Authors suggested several explanations for this finding that should be further explored, such as changes on olfactory functioning over time, the exact odour exposure time and habituation effect. One study measured the impact of olfactory stimulation on balance and reported a significant effect following prolonged exposure to the olfactory stimulus. Overall, the encouraging results found on physical functioning suggest that there continues to be a need for further research to assess the effect of olfactory stimulation in relation to these areas.

The various outcomes reported were evaluated through quantitative research design, which surprisingly constituted the only sources of evidence of olfactory stimulation in dementia care. While quantitative research is particularly useful to quantify intervention effects, there are some limitations to consider when applied in dementia research on olfactory intervention. For example, quantitative methods do not enable capture of participants’ perspectives on the nature of change and positive effects that are likely to occur ‘in the moment’ at the verbal and non-verbal level (Webb et al., 2020). Furthermore, as most people with severe dementia might present communication difficulties, observational measures may be helpful to explore the individual’s experience and investigate the potential role of olfactory stimuli in dementia care. Qualitative or mixed methods designs could extend the quantitative findings by offering insights into participants’ olfactory experiences and the specific features associated with olfactory stimulation in dementia care. Non-invasive physiological measures such as skin conductance or cardiovascular response using new technologies and instruments could also provide valuable information on physiological responses when people are less able to communicate their subjective affective experience (Garbarino et al., 2014).

The included studies varied greatly in terms of administration methods, procedures and outcomes. The high heterogeneity found in the intervention protocols and the limited number of studies for each outcome (i.e. sleep, food intake, cognitive function and balance) made comparisons between the studies difficult.

Attempts were made to identify patterns between olfactory outcomes and the different approaches used to administer olfactory stimuli among the studies. Among the three studies that did not report significant improvements in responsive behaviour, scent-infused fabric or body oil spray were used. These application methods might be associated with habituation effects, that is, a decrease in individual’s response due to the continual exposure to an odour. Other factors such as the source, grade and dilution of olfactory stimuli, and the method of administration might have potential implications for the success of the interventions. In this review, inferences regarding the habituation effect and odour concentration on the outcomes could not be made due to the limited information reported by the included studies.

It remains unclear if other factors such as the number of sessions and the length of interventions might play a role in the reported outcomes. Repeated and prolonged scent exposure (e.g. every day for almost 1 year) was associated with positive outcomes. There is evidence showing that olfactory sensitivity is greatly reduced following constant exposure, that is, over 20 minutes, and dramatically diminished if the odour is encountered throughout the day (Dalton & Wysocki, 1996; Stuck et al., 2014). On the other hand, there is evidence that olfactory stimuli at subthreshold levels (unconscious perception) may influence behaviours and responses to the surrounding environment (Dalton et al. 2000). Further research is needed to draw firm conclusions about the most appropriate smell administration methods for people with dementia and to identify other factors influencing the outcomes.

Regarding olfactory stimuli, the majority of studies used lavender oil to reduce responsive behaviours. Lavender has a long history of medical use and has been employed for its sedative and calming properties (Cavanagh & Wilkinson, 2002; Sayorwan et al., 2012). Although it has been widely used in olfactory stimulation, specific pharmacological effects of lavender aromatherapy are difficult to distinguish from any innate or learned preference for this scent (Bradley et al., 2009). This may also explain the mixed results observed in the studies included.

Among the studies included only three considered the participants’ smell preferences and familiarity. Smell preference and past experience create the framework upon which response to odour takes place (Herz, 2016). This is particularly relevant in the context of triggering autobiographic memories or behaviour change. Inter-individual characteristics can modulate the degree to which scents elicit responses. Therefore, it could be expected that stage and subtype of dementia as well as individual olfactory function may influence the outcomes. The majority of the studies included people with AD and VaD. There were very few studies involving people with FTD and DLB; so, there is currently limited evidence as to what extent olfactory stimulation may be useful for these groups and whether the subtype of dementia could be relevant to outcomes.

Although people with dementia may present an impaired sense of smell, only eight studies assessed the participants’ olfactory functioning. It was therefore unclear to what extent participants had an olfactory experience or indeed if they could perceive the odours at all. Olfactory screening at baseline can increase certainty that the participants are able to perceive the smells presented. Standardised screening tests lasting approximately 40 min, such as smell identification tasks, might present practical limitations when used with people with dementia who often present communication difficulties (e.g. aphasia) and a limited ability to focus and maintain concentration for an extended period, especially in the later stages of the condition. Recording participants’ verbal and non-verbal reactions to smells presents an alternative way to screen the olfactory functioning of people with dementia. However, due to the large inter-individual variability of people’s responses, this method might lack standardisation. Future studies should include olfactory screening investigating the best method to conduct it.

The encouraging results found in the present review suggest that olfactory exposure might be considered a potentially effective non-pharmacological intervention for people with dementia and indicates a direction for future research. Due to the limited body of evidence, the methodological limitations and the diversity of approaches used, it was not possible to draw any clear conclusion about the efficacy of olfactory stimulation.

### Strengths and limitations

The review has benefitted from the inclusion of a range of study designs and methods that provided an overview of the field and a rich source of data on olfactory interventions and their effects. In addition, excluding the studies using olfactory stimuli in combination with other sensory activities or massage reduced the risk that any positive findings identified could be related to variables other than olfactory stimulation.

There are some limitations in this review. Only studies published in English were included. While there were positives to a single-reviewer approach with verification of a subset of articles by the second reviewer, such as reducing the time and streamlining the review process, this approach may leave the review open to bias and errors. The inclusion criteria could have neglected some important information. In particular, the decision to exclude studies that used touch or massage alongside olfactory stimuli was made to exclude contamination of purely olfactory effects by tactile stimulation. However, doing so means that this review could not explore the interaction of different forms of olfactory stimulation. Finally, publication bias could have affected the overall conclusions. It is recognised that studies with negative results are often unreported, which consequently may misinform the review’s conclusions (Mlinarić et al., 2017).

### Recommendations

Future research should systematically investigate the conflicting outcomes reported, by clarifying why and how olfactory stimulation works. To this aim, high methodological quality of studies and detailed research protocols are required to allow examination of similarities and differences and to compare effects. Qualitative investigations are essential to provide a further insight into the experience of olfactory stimulation and any factors associated with positive outcomes.

Previous experience, preference and cultural context play a relevant role in how people perceive odours and in predicting individual’s responses. Therefore, further studies should take these factors into account (Herz, 2016). Finally, olfactory stimulation effects on those with different types of dementia should be investigated. This is because dementias affect the olfactory system differently (Alves et al., 2014), and olfactory stimulation effects would be expected to differ.

## Conclusion

Despite the heterogeneity of methods in the included papers, the results of the studies are generally in favour of the use of olfactory stimulation. Olfactory intervention in dementia care is an emergent area of research warranting attention since current data suggests that smells may promote physical health, cognitive and behavioural changes, with minimal or no adverse events (Ball et al., 2020). Given that smells trigger positive emotional and autobiographic memories, olfactory stimulation might be useful to improve the quality of life and well-being of people with dementia and those who care for them.

## Supplemental Material

sj-pdf-1-dem-10.1177_14713012221082377 – Supplemental Material for Olfactory stimulation for people with dementia: A rapid review Click here for additional data file. Supplemental Material, sj-pdf-1-dem-10.1177_14713012221082377 for Olfactory stimulation for people with dementia: A rapid review by Federica D’Andrea, Victoria Tischler, Tom Dening and Anne Churchill in Dementia

## References

[bibr1-14713012221082377] Alves J. Petrosyan A. Magalhães R. (2014). Olfactory dysfunction in dementia. World Journal of Clinical Cases, 2(11), 661–667. 10.12998/wjcc.v2.i11.661 25405189 PMC4233420

[bibr2-14713012221082377] Arruda M. Viana H. Rainha N. Neng N. R. Rosa J. S. Nogueira J. M. F. Do Carmo Barreto M. (2012). Anti-acetylcholinesterase and antioxidant activity of essential oils from Hedychium gardnerianum sheppard ex ker-gawl. Molecules: A Journal of Synthetic Chemistry and Natural Product Chemistry, 17(3), 3082–3092. 10.3390/molecules17033082 22410418 PMC6268484

[bibr3-14713012221082377] Arshamian A. Iannilli E. Gerber J. C. Willander J. Persson J. Seo H. S. Hummel T. Larsson M. (2013). The functional neuroanatomy of odor evoked autobiographical memories cued by odors and words. Neuropsychologia, 51(1), 123–131. 10.1016/j.neuropsychologia.2012.10.023 23147501

[bibr4-14713012221082377] Attems J. Walker L. Jellinger K. A. (2014). Olfactory bulb involvement in neurodegenerative diseases. Acta Neuropathologica, 127(4), 459–475. 10.1007/s00401-014-1261-7 24554308

[bibr5-14713012221082377] Ball E. L. Owen-Booth B. Gray A. Shenkin S. D. Hewitt J. McCleery J. (2020). Aromatherapy for dementia. Cochrane Database of Systematic Reviews, 8(8), CD003150. 10.1002/14651858.CD003150.pub3 32813272 PMC7437395

[bibr6-14713012221082377] D’Andrea F. Victoria T. Tom D. Anne C. Esther H . (2020). The effects of olfactory stimuli on people with dementia: a rapid review of the literature. PROSPERO. CRD42020202670

[bibr7-14713012221082377] Bradley B. F. Brown S. L. Chu S. Lea R. W. (2009). Effects of orally administered lavender essential oil on responses to anxiety-provoking film clips. Human Psychopharmacology: Clinical and Experimental, 24(4), 319–330. 10.1002/hup.1016 19382124

[bibr8-14713012221082377] Brooker D. J. R. Snape M. Johnson E. Ward D. Payne M. (1997). Single case evaluation of the effects of aromatherapy and massage on disturbed behaviour in severe dementia. British Journal of Clinical Psychology, 36(2), 287–296. 10.1111/j.2044-8260.1997.tb01415.x 9167869

[bibr9-14713012221082377] Cavanagh H. M. A. Wilkinson J. M. (2002). Biological activities of lavender essential oil. Phytotherapy Research, 16(4), 301–308. 10.1002/ptr.1103 12112282

[bibr10-14713012221082377] Chu S. Downes J. J. (2000). Odour-evoked autobiographical memories: Psychological investigations of proustian phenomena. Chemical Senses, 25(1), 111–116. 10.1093/chemse/25.1.111 10668001

[bibr11-14713012221082377] Cohen-Mansfield J. Kerin P. (1986). Agitation in nursing home elderly: A quantitative development of the concept. Monograph.

[bibr12-14713012221082377] Cummings J. L. Mega M. Gray K. Rosenberg-Thompson S. Carusi D. A. Gornbein J. (1994). The neuropsychiatric inventory: Comprehensive assessment of psychopathology in dementia. Neurology, 44(12), 2308–2314. 10.1212/wnl.44.12.2308 7991117

[bibr13-14713012221082377] Dalton P. Doolittle N. Nagata H. Breslin P. A. S. (2000). The merging of the senses: integration of subthreshold taste and smell. Nature Neuroscience, 3(5), 431–432. 10.1038/74797 10769380

[bibr14-14713012221082377] Dalton P. Wysocki C. J. (1996). The nature and duration of adaptation following long-term odor exposure. Perception and Psychophysics, 58(5), 781–792. 10.3758/BF03213109 8710455

[bibr15-14713012221082377] Degel J. Köster E. P. (1999). Odors: Implicit memory and performance effects. Chemical Senses, 24(3), 317–325. 10.1093/chemse/24.3.317 10400450

[bibr16-14713012221082377] Degel J. Piper D. Köster E. P. (2001). Implicit learning and implicit memory for odors: The influence of odor identification and retention time. Chemical Senses, 26(3), 267–280. 10.1093/chemse/26.3.267 11287387

[bibr17-14713012221082377] Doty R. L. Kamath V. (2014). The influences of age on olfaction: a review. Frontiers in Psychology, 5, 20. 10.3389/fpsyg.2014.00020 24570664 PMC3916729

[bibr18-14713012221082377] El Haj M. Gandolphe M. C. Gallouj K. Kapogiannis D. Antoine P. (2018). From nose to memory: The involuntary nature of odor-evoked autobiographical memories in Alzheimer’s disease. Chemical Senses, 43(1), 27–34. 10.1093/chemse/bjx064 PMC5863564 29040475

[bibr19-14713012221082377] Fleischman D. A. Wilson R. S. Gabrieli J. D. E. Schneider J. A. Bienias J. L. Bennett D. A. (2005). Implicit memory and Alzheimer’s disease neuropathology. Brain: a Journal of Neurology, 128(9), 2006–2015. 10.1093/brain/awh559 15975947

[bibr20-14713012221082377] Freeman S. Ebihara S. Ebihara T. Niu K. Kohzuki M. Arai H. Butler J. P. (2009). Olfactory stimuli and enhanced postural stability in older adults. Gait and Posture, 29(4), 658–660. 10.1016/j.gaitpost.2009.02.005 19286382

[bibr21-14713012221082377] Fu C. Y. Moyle W. Cooke M. (2013). A randomised controlled trial of the use of aromatherapy and hand massage to reduce disruptive behaviour in people with dementia. BMC Complementary and Alternative Medicine, 13(1), 1–9. 10.1186/1472-6882-13-165 23837414 PMC3737022

[bibr22-14713012221082377] Fung J. K. K. Tsang H. W. H. Chung R. C. K. (2012). A systematic review of the use of aromatherapy in treatment of behavioral problems in dementia. Geriatrics and Gerontology International, 12(3), 372–382. 10.1111/j.1447-0594.2012.00849.x 22433025

[bibr23-14713012221082377] Garbarino M. Lai M. Bender D. Picard R. W. Tognetti S. (2014). Empatica E3—A wearable wireless multi-sensor device for real-time computerized biofeedback and data acquisition. In 2014 4th International Conference on Wireless Mobile Communication and Healthcare-Transforming Healthcare Through Innovations in Mobile and Wireless Technologies (MOBIHEALTH) (pp. 39–42). IEEE.

[bibr24-14713012221082377] Glachet O. El Haj M. (2019). Emotional and phenomenological properties of odor-evoked autobiographical memories in Alzheimer’s disease. Brain Sciences, 9(6), 135. 10.3390/brainsci9060135 PMC6627121 31185649

[bibr25-14713012221082377] Glachet O. El Haj M. (2020a). Effects of olfactory stimulation on past and future thinking in Alzheimer’s disease. Chemical Senses, 45(4), 313–320. 10.1093/chemse/bjaa016 32157274

[bibr26-14713012221082377] Glachet O. El Haj M. (2020b). Smell your self: Olfactory stimulation improves self-concept in Alzheimer’s disease. Neuropsychological Rehabilitation, 0(0), 1–17. 10.1080/09602011.2020.1831553 33078674

[bibr27-14713012221082377] Glachet O. Moustafa A. A. Gallouj K. El Haj M. (2019). Smell your memories: Positive effect of odor exposure on recent and remote autobiographical memories in Alzheimer’s disease. Journal of Clinical and Experimental Neuropsychology, 41(6), 555–564. 10.1080/13803395.2019.1586840 30890017

[bibr28-14713012221082377] Gray S. G. Clair A. A. (2002). Influence of aromatherapy on medication administration to residential-care residents with dementia and behavioral challenges. American Journal of Alzheimer’s Disease and Other Dementias, 17(3), 169–174. 10.1177/153331750201700305 PMC10833692 12083347

[bibr29-14713012221082377] Hamel C. Michaud A. Thuku M. Skidmore B. Stevens A. Nussbaumer-Streit B. Garritty C. (2021). Defining rapid reviews: a systematic scoping review and thematic analysis of definitions and defining characteristics of rapid reviews. Journal of Clinical Epidemiology, 129, 74–85. 10.1016/j.jclinepi.2020.09.041 33038541

[bibr30-14713012221082377] Hansen N. V. Jørgensen T. Ørtenblad L. (2006). Massage and touch for dementia. Cochrane Database of Systematic Reviews, 2006(4), CD004989. 10.1002/14651858.CD004989.pub2 PMC6823223 17054228

[bibr31-14713012221082377] Henry J. Rusius C. W. Davies M. Veazey-French T. (1994). Lavender for night sedation of people with dementia. International Journal of Aromatherapy, 5(2), 28–30.

[bibr32-14713012221082377] Herz R. S. (1998). Are odors the best cues to memory? Annals of the New York Academy of Sciences, 855(1), 670–674. 10.1111/j.1749-6632.1998.tb10643.x 9929669

[bibr33-14713012221082377] Herz R. (2016). The role of odor-evoked memory in psychological and physiological health. Brain Sciences, 6(3), 22. 10.3390/brainsci6030022 PMC5039451 27447673

[bibr34-14713012221082377] Herz R. S. Eliassen J. Beland S. Souza T. (2004). Neuroimaging evidence for the emotional potency of odor-evoked memory. Neuropsychologia, 42(3), 371–378. 10.1016/j.neuropsychologia.2003.08.009 14670575

[bibr35-14713012221082377] Holmes C. Hopkins V. Hensford C. MacLaughlin V. Wilkinson D. Rosenvinge H. (2002). Lavender oil as a treatment for agitated behaviour in severe dementia: A placebo controlled study. International Journal of Geriatric Psychiatry, 17(4), 305–308. 10.1002/gps.593 11994882

[bibr36-14713012221082377] Homma A. Niina R. Ishii T. Hasegawa K. (1991). Behavioral evaluation of Alzheimer disease in clinical trials: Development of the Japanese version of the GBS scale. Alzheimer Disease and Associated Disorders, 5(Suppl 1), S40–S48. 10.1097/00002093-199100051-00008 1781973

[bibr37-14713012221082377] Hong Q. N. Pluye P. Fàbregues S. Bartlett G. Boardman F. Cargo M. Dagenais P. Gagnon M. P. Griffiths F. Nicolau B. Rousseau M. C. Vedel I. (2018). Mixed methods appraisal tool (MMAT) version 2018 User guide. Mixed Methods Appraisal Tool Public. http://mixedmethodsappraisaltoolpublic.pbworks.com/10.1016/j.jclinepi.2019.03.008 30905698

[bibr38-14713012221082377] Hui E.K. Tischler V. Wong G.H.Y. Lau W.Y.T. Spector A. (2021). Systematic review of the current psychosocial interventions for people with moderate to severe dementia. International Journal of Geriatric Psychiatry, 36(9), 1313–1329. 10.1002/gps.5554 34350626

[bibr39-14713012221082377] Inoue M. Jimbo D. Taniguchi M. Urakami K. (2011). Touch panel-type dementia assessment scale: A new computer-based rating scale for Alzheimer’s disease. Psychogeriatrics: The Official Journal of the Japanese Psychogeriatric Society, 11(1), 28–33. 10.1111/j.1479-8301.2010.00345.x 21447106

[bibr40-14713012221082377] Jimbo D. Kimura Y. Taniguchi M. Inoue M. Urakami K. (2009). Effect of aromatherapy on patients with Alzheimer’s disease. Psychogeriatrics: The Official Journal of the Japanese Psychogeriatric Society, 9(4), 173–179. 10.1111/j.1479-8301.2009.00299.x 20377818

[bibr41-14713012221082377] Kim E. K. Park H. Lee C. H. Park E. (2019). Effects of aromatherapy on agitation in patients with dementia: A systematic literature review and meta-analysis. Journal of Korean Academy of Community Health Nursing, 30(2), 183–194. 10.12799/jkachn.2019.30.2.183

[bibr42-14713012221082377] Leung V. P. Y. Lam L. C. W. Chiu H. F. K. Cummings J. L. Chen Q. L. (2001). Validation study of the Chinese version of the neuropsychiatric inventory (CNPI). International Journal of Geriatric Psychiatry, 16(8), 789–793. 10.1002/gps.427 11536346

[bibr43-14713012221082377] Lin P. W. K. Chan W. C. Ng B. F. L. Lam L. C. W. (2007). Efficacy of aromatherapy (Lavandula angustifolia) as an intervention for agitated behaviours in Chinese older persons with dementia: A cross-over randomized trial. International Journal of Geriatric Psychiatry, 22(5), 405–410. 10.1002/gps.1688 17342790

[bibr44-14713012221082377] Lis-Balchin M. Hart S. (1999). Studies on the mode of action of the essential oil of lavender (Lavandula angustifolia P. Miller). Phytotherapy Research, 13(6), 540–542. 10.1002/(SICI)1099-1573(199909)13:6<540::AID-PTR523>3.0.CO;2-I 10479772

[bibr45-14713012221082377] Lopis D. Pape T. L. Manetta C. Conty L. (2021). Sensory cueing of autobiographical memories in normal aging and Alzheimer’s disease: A comparison between visual, auditory, and olfactory information. Journal of Alzheimer’s Disease, 80(3), 1169–1183. https://doi.org10.3233/JAD-200841 10.3233/JAD-200841 PMC8150461 33646149

[bibr46-14713012221082377] Mlinarić A. Horvat M. Smolčić V. Š. (2017). Dealing with the positive publication bias: Why you should really publish your negative results. Biochemia Medica, 27(3), 30201. 10.11613/BM.2017.030201 PMC5696751 29180912

[bibr47-14713012221082377] Moher D. Liberati A. Tetzlaff J. Altman D. G. (2009). Preferred reporting items for systematic reviews and meta-analyses: The PRISMA statement. PLoS Medicine, 6(7), Article e1000097. 10.1371/journal.pmed.1000097 19621072 PMC2707599

[bibr48-14713012221082377] Moorman Li R. Gilbert B. Orman A. Aldridge P. Leger-Krall S. Anderson C. Hincapie Castillo J. (2017). Evaluating the effects of diffused lavender in an adult day care center for patients with dementia in an effort to decrease behavioral issues: A pilot study. Journal of Drug Assessment, 6(1), 1–5. 10.1080/21556660.2016.1278545 28265482 PMC5327916

[bibr49-14713012221082377] Murphy C. (2019). Olfactory and other sensory impairments in Alzheimer disease. Nature Reviews Neurology, 15(1), 11–24. 10.1038/s41582-018-0097-5 30532084

[bibr51-14713012221082377] Ouzzani M. Hammady H. Fedorowicz Z. Elmagarmid A. (2016). Rayyan-a web and mobile app for systematic reviews. Systematic Reviews, 5(1), 210. 10.1186/s13643-016-0384-4 27919275 PMC5139140

[bibr52-14713012221082377] Park H. Chun Y. Kwak S. (2016). The effects of aroma hand massage on fatigue and sleeping among hospice patients. Open Journal of Nursing, 6(7), 515–523. 10.4236/ojn.2016.67054

[bibr53-14713012221082377] Pouliot S. Jones-Gotman M. (2008). Medial temporal-lobe damage and memory for emotionally arousing odors. Neuropsychologia, 46(4), 1124–1134. 10.1016/j.neuropsychologia.2007.10.017 18054971

[bibr54-14713012221082377] Saito S. Ayabe-Kanamura S. Takashima Y. Gotow N. Naito N. Nozawa T. Mise M. Deguchi Y. Kobayakawa T. (2006). Development of a smell identification test using a novel stick-type odor presentation kit. Chemical Senses, 31(4), 379–391. 10.1093/chemse/bjj042 16527871

[bibr55-14713012221082377] Sakamoto Y. Ebihara S. Ebihara T. Tomita N. Toba K. Freeman S. Arai H. Kohzuki M. (2012). Fall prevention using olfactory stimulation with lavender odor in elderly nursing home residents: A randomized controlled trial. Journal of the American Geriatrics Society, 60(6), 1005–1011. 10.1111/j.1532-5415.2012.03977.x 22646853

[bibr56-14713012221082377] Sayorwan W. Siripornpanich V. Piriyapunyaporn T. Hongratanaworakit T. Kotchabhakdi N. Ruangrungsi N. (2012). The effects of lavender oil inhalation on emotional states, autonomic nervous system, and brain electrical activity. Journal of the Medical Association of Thailand, 95(4), 598–606. https://pubmed.ncbi.nlm.nih.gov/22612017/22612017

[bibr57-14713012221082377] Smallwood J. Brown R. Coulter F. Irvine E. Copland C. (2001). Aromatherapy and behaviour disturbances in dementia: A randomized controlled trial. International Journal of Geriatric Psychiatry, 16(10), 1010–1013. 10.1002/gps.473 11607948

[bibr58-14713012221082377] Snow A. L. Hovanec L. Brandt J. (2004). A controlled trial of aromatherapy for agitation in nursing home patients with dementia. Journal of Alternative and Complementary Medicine, 10(3), 431–437. 10.1089/1075553041323696 15253846

[bibr59-14713012221082377] Sowndhararajan K. Kim S. (2016). Scientia pharmaceutica influence of fragrances on human psychophysiological activity: With special reference to human electroencephalographic response. Scientia Pharmaceutica, 84(4), 724–751. 10.3390/scipharm84040724 27916830 PMC5198031

[bibr60-14713012221082377] Stuck B. A. Fadel V. Hummel T. Ulrich Sommer J. (2014). Subjective olfactory desensitization and recovery in humans. Chemical Senses, 39(2), 151–157. 10.1093/chemse/bjt064 24293565

[bibr61-14713012221082377] Sulmont-Rossé C. Gaillet M. Raclot C. Duclos M. Servelle M. Chambaron S. (2018). Impact of olfactory priming on food intake in an Alzheimer’s disease unit. Journal of Alzheimer’s Disease, 66(4), 1497–1506. 10.3233/JAD-180465 30452411

[bibr62-14713012221082377] Takahashi Y. Shindo S. Kanbayashi T. Takeshima M. Imanishi A. Mishima K. (2020). Examination of the influence of cedar fragrance on cognitive function and behavioral and psychological symptoms of dementia in Alzheimer type dementia. Neuropsychopharmacology Reports, 40(1), 10–15. 10.1002/npr2.12096 32037737 PMC7292212

[bibr63-14713012221082377] Takeda A. Watanuki E. Koyama S. (2017). Effects of inhalation aromatherapy on symptoms of sleep disturbance in the elderly with dementia. Evidence-Based Complementary and Alternative Medicine, 2017(4), Article 1902807. 10.1155/2017/1902807 28400839 PMC5376423

[bibr64-14713012221082377] Tricco A. C. Langlois E. V. Straus S. E. (2017). Rapid reviews to strengthen health policy and systems: A practical guide. World Health Organization. http://www.who.int/alliance-hpsr/resources/publications/rapid-review-guide/en/

[bibr65-14713012221082377] Webb J. Williams V. Gall M. Dowling S. (2020). Misfitting the research process: Shaping qualitative research “in the field” to fit people living with dementia. International Journal of Qualitative Methods, 19, 1-11, 10.1177/1609406919895926

[bibr66-14713012221082377] White T. L. Møller P. Köster E. P. Eichenbaum H. Linster C. (2015). Olfactory Memory. In Handbook of olfaction and gustation (pp. 337–352). John Wiley & Sons, Inc. 10.1002/9781118971758.ch15

[bibr68-14713012221082377] Willander J. Larsson M. (2007). Olfaction and emotion: The case of autobiographical memory. Memory and Cognition, 35(7), 1659–1663. 10.3758/BF03193499 18062543

[bibr69-14713012221082377] World Health Organization . (2020). Dementia: Key facts. WHO. https://www.who.int/news-room/fact-sheets/detail/dementia

[bibr70-14713012221082377] Zoon H. de Graaf C. Boesveldt S. (2016). Food odours direct specific appetite. Foods, 5(1), 12. 10.3390/foods5010012 PMC5224573 28231107

