# Supplementary material for: Olfactory stimulation for people with dementia: A rapid review
Source: Dementia (London). 2022 Apr 17;21(5):1800–24. doi: 10.1177/14713012221082377 (PMC9243450; doi:10.1177/14713012221082377)
Supplement: sj-pdf-1-dem-10.1177_14713012221082377 – Supplemental Material for Olfactory stimulation for people with dementia: A rapid review [file sj-pdf-1-dem-10.1177_14713012221082377.pdf]

Supplementary materials

Figure S1. Summary of risk of bias in studies.
The three figures summary the review authors' judgements about each risk of bias item for all included study in the appropriate Mixed Methods Appraisal Tool (MMAT) category: (a) randomised controlled trial studies; (b) quantitative descriptive study (c) quantitative non-randomised studies.

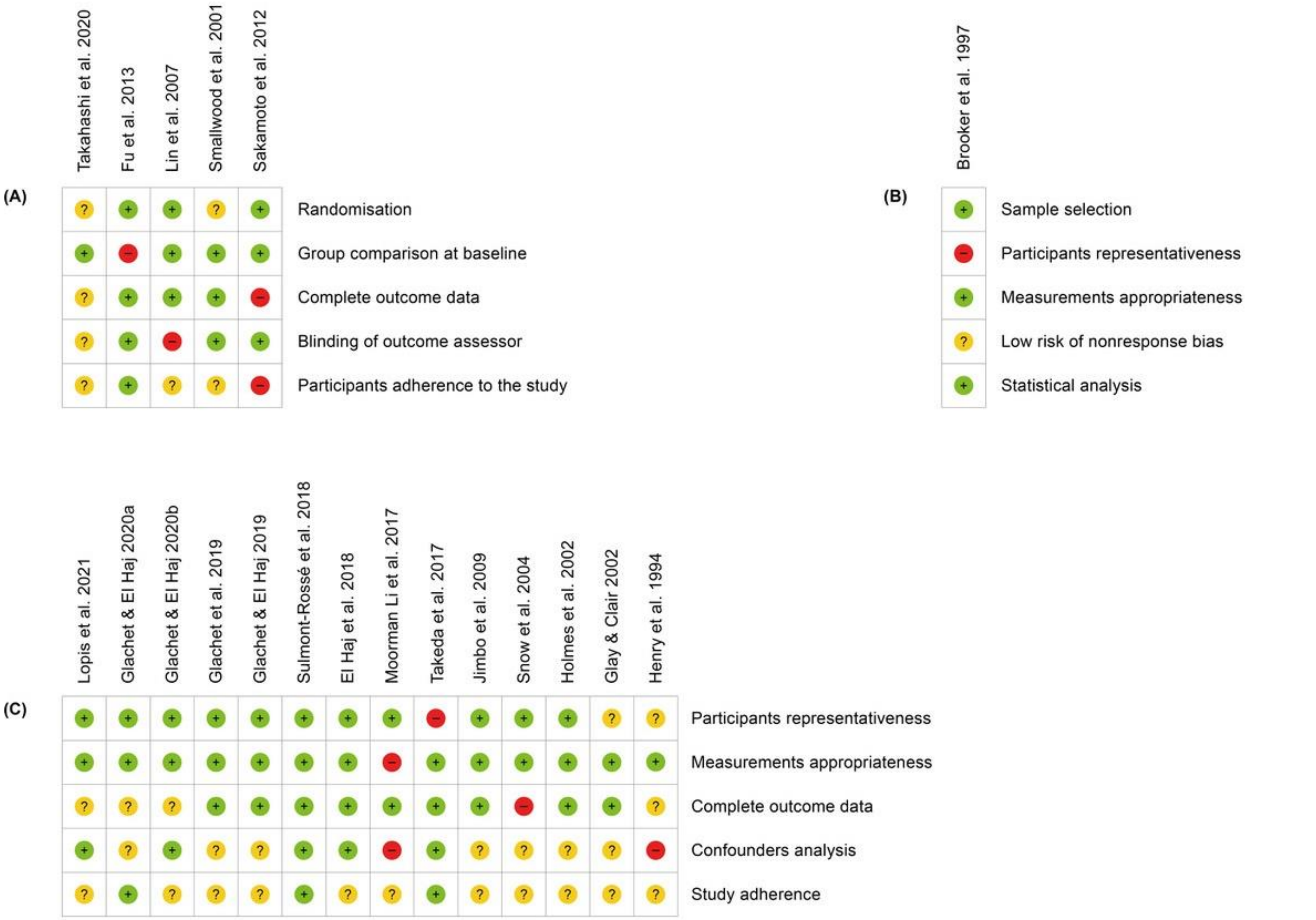

Table S1. Inclusion and exclusion criteria.

**Inclusion criteria**	**Exclusion criteria**
Studies of any design, using quantitative, qualitative, or mixed methods that reported the results of olfactory stimulation for people with dementia.	Study only reported on the olfactory function of the participants rather than the effects of the stimuli.
Study compared purely olfactory stimulation intervention with other interventions such as those combining massage with essential oils.	Studies of aromatherapy using massage or touch, multi-sensory intervention, Sonas programme, Namaste Care programme and any study combining olfactory stimulation with other activities.
Study must be conducted with people with dementia even if specific diagnoses were not provided. No specific restrictions regarding age, subtype and severity of dementia.	Unpublished papers, study protocols, dissertations and review papers.
Study must use olfactory stimuli.	Studies not in English language.
Study must report the effects of olfactory stimuli on the participants.	

Table S2. Summary of search terms.

**Search**	**Terms**
#1	Dement* OR alzheim* OR mixed dementia* OR vascular dementia OR Lewy Body
#2	olfactory OR smell OR scent OR perfume OR odor* OR odour* OR aroma*
#3	intervention OR activit* OR session OR reminisc* OR memor* OR experienc*
#4	dysfunction OR impairment
#5	#1 AND #2 AND #3 NOT #4

Table S3. Preferred Reporting Items for Systematic Reviews and Meta-Analyses (PRISMA) statement (Moher et al., 2009) integrated with a list of key reporting items for rapid review (Tricco et al., 2017).

Section/topic	#	Checklist item	Reported on page #
**TITLE**			
Title	1	Identify the report as a rapid review*	p. 1
**ABSTRACT**			
Structured summary	2	Provide a structured summary including, as applicable: background; objectives; data sources; study eligibility criteria, participants, and interventions; study appraisal and synthesis methods; results; limitations; conclusions and implications of key findings; systematic review registration number.	p. 1
**INTRODUCTION**			
Rationale	3	Describe the rationale for the review in the context of what is already known.	pp. 1-3
Objectives	4	Provide an explicit statement of questions being addressed with reference to participants, interventions, comparisons, outcomes, and study design (PICOS).	p. 3
**METHODS**			
Protocol and registration	5	Indicate if a review protocol exists, if and where it can be accessed (e.g., Web address), and, if available, provide registration information including registration number.	p. 3
Eligibility criteria	6	Specify study characteristics (e.g., PICOS, length of follow-up) and report characteristics (e.g., years considered, language, publication status) used as criteria for eligibility, giving rationale.<br>Indicate if limits on the types of study designs included were applied (e.g. existing systematic reviews, randomized controlled trials).*<br>Specify if the search strategy was limited in any way (e.g. number of databases, grey literature, date, setting, language).*	pp. 3-4
Information sources	7	Describe all information sources (e.g., databases with dates of coverage, contact with study authors to identify additional studies) in the search and date last searched.	pp. 4-6
Search	8	Present full electronic search strategy for at least one database, including any limits used, such that it could be repeated.	p. 4 (Table 2)
Study selection	9	State the process for selecting studies (i.e., screening, eligibility, included in systematic review, and, if applicable, included in the meta-analysis).<br>Indicate if the process of dual study selection was modified or omitted.*	pp. 4-5
Data collection process	10	Describe method of data extraction from reports (e.g., piloted forms, independently, in duplicate) and any processes for obtaining and confirming data from investigators.<br>Indicate if the process of dual data extraction was modified or omitted.*	pp. 5-6
Data items	11	List and define all variables for which data were sought (e.g., PICOS, funding sources) and any assumptions and simplifications made.	pp. 6-7
Risk of bias in individual studies	12	Describe methods used for assessing risk of bias of individual studies (including specification of whether this was done at the study or outcome level), and how this information is to be used in any data synthesis.	p. 5-6

		Specify if the assessment of risk of bias or quality of evidence was limited or omitted.*	
Summary measures	13	State the principal summary measures (e.g., risk ratio, difference in means).	p. 6-7
Synthesis of results	14	Describe the methods of handling data and combining results of studies, if done, including measures of consistency (e.g., I^2) for each meta-analysis.<br>Describe if the qualitative or quantitative analysis was limited or omitted*.	p. 6-7
Risk of bias across studies	15	Specify any assessment of risk of bias that may affect the cumulative evidence (e.g., publication bias, selective reporting within studies).	-
Additional analyses	16	Describe methods of additional analyses (e.g., sensitivity or subgroup analyses, meta-regression), if done, indicating which were pre-specified.	-
**RESULTS**			
Study selection	17	Give numbers of studies screened, assessed for eligibility, and included in the review, with reasons for exclusions at each stage, ideally with a flow diagram.	p. 7
Study characteristics	18	For each study, present characteristics for which data were extracted (e.g., study size, PICOS, follow-up period) and provide the citations.	pp. 7-18
Risk of bias within studies	19	Present data on risk of bias of each study and, if available, any outcome level assessment (see item 12).	pp. 18-20
Results of individual studies	20	For all outcomes considered (benefits or harms), present, for each study: (a) simple summary data for each intervention group (b) effect estimates and confidence intervals, ideally with a forest plot.	pp. 7-18
Synthesis of results	21	Present results of each meta-analysis done, including confidence intervals and measures of consistency.	-
Risk of bias across studies	22	Present results of any assessment of risk of bias across studies (see Item 15).	-
Additional analysis	23	Give results of additional analyses, if done (e.g., sensitivity or subgroup analyses, meta-regression [see Item 16]).	-
**DISCUSSION**			
Summary of evidence	24	Summarize the main findings including the strength of evidence for each main outcome; consider their relevance to key groups (e.g., healthcare providers, users, and policy makers).	pp. 20-25
Limitations	25	Discuss limitations at study and outcome level (e.g., risk of bias), and at review-level (e.g., incomplete retrieval of identified research, reporting bias).<br>Provide a disclaimer and/or limitations section in context with your findings, if appropriate.*	p. 25
Conclusions	26	Provide a general interpretation of the results in the context of other evidence, and implications for future research.	p. 26
**FUNDING**			
Funding	27	Describe sources of funding for the systematic review and other support (e.g., supply of data); role of funders for the systematic review.	p. 27

* Suggested minimum reporting items for rapid reviews of health policy and systems research (Tricco et al., 2017).

Table S4. Overview of the olfactory stimuli and administration methods.

Domain	Study	Scents	Concentration	Administration method & Dosage
Autobiographical memory	Lopis et al. (2021)	Apple	-	Inhalation - sniffing sticks
		Coffee		
		Fresh-cut grass		
		Laundry		
	Glachet & El Haj (2020a) Glachet & El Haj (2020b)	Peach	-	Inhalation - bottles scented oil
		Orange		
		Grass		
		Cinnamon		
		Chocolate		
		Coffee		
		Coconut		
	Glachet et al. (2019)	Coffee	-	Inhalation - bottles scented oil
	Glachet & El Haj (2019)	Cinnamon	-	Inhalation - bottles scented oil
	El Haj et al. (2018)	Coffee	-	Inhalation - bottles scented oil
		Vanilla		
Responsive behaviour	Takahashi et al. (2020)	Ethanol with cedar leaves	20 gr of cedar leaves cut into 1 cm strips and added to 200 ml of a 20% ethanol solution. This solution was distilled to 50% ethanol at 60°C under a lowered pressure (170 hpa) with a rotary evaporator.	Inhalation & Spray - wood (i.e. rattan) sticks (2.3 ml of distilled liquid delivered per day at room temperature) and spray type onto clothing and bedding a few times a day
	Moorman Li et al. (2017)	Lavender		Inhalation - diffuser<br><br>Diffuser in 1000 square feet in size and in a moderately open space for 20 min twice a day, once in the morning and once in the mid-afternoon. The estimated oil output ranges from 0.75 to 1.3 ml over 15 minutes.

	Lin et al. (2007)	Lavender (*Lavandula angustifolia*)<br><br>Sunflower (control condition)	Pure undiluted lavender	Inhalation - two diffusers<br><br>A cotton pad with two drops of essential oil placed in each of the two diffusers positioned at each side of participant's pillow during sleep at night for at least 1 h
	Smallwood et al. (2001)	Lavender	-	Inhalation - diffuser
	Fu et al. (2013)	Lavender (*Lavandula angustifolia*)	A 3% lavender mist, consisting of 75 drops of pure 100% lavender oil was mixed with 4 ml essential oil solubiliser and 125 cc purified water	Spray - direct spray onto individuals' upper chest within a 30 cm distance
	Snow et al. (2004)	Lavender (*Lavandula angustifolia*)	Pure undiluted oils	Fabric - sachet<br>Two drops of pure undiluted oil placed on a 2 x 2-inch absorbent fabric sachet pinned to the front of each participant's shirt near the collarbone, every 3 hours for a total of three applications per day
		Thyme oil (*Thymus vulgaris*)		
		Unscented grapeseed oil		
	Gray & Clair (2002)	Tea tree (*Malaleuca alternifolia*)	-	Fabric - cotton-ball placed over the mouth of a four-ounce oil bottle, and the bottle was inverted completely for no more than two seconds before it was returned to the upright position. The cotton-ball taped to the lapel of resident
		Sweet orange (*Citrus aurantium*)		
		Lavender (*Lavendulan officinalis*)		
	Holmes et al. (2002)	Lavender	2% lavender	Inhalation - three aroma steam diffusers
	Brooker et al. (1997)	Lavender		Inhalation – fan
Physical function	Sakamato et al. (2012)	Lavender	-	Paper patch (size: 1 cm x 2 cm)<br>attached to the inside of the resident's clothes near the neck for 24 hours for 360 days

Eating behaviour	Sulmont-Rossé et al. (2018)	Meat odour “sauté de boeuf” (lit. “Beef stir-fry”)	-	Inhalation - diffusers<br><br>Distributing in the room 90-s puffs every 30-s for the large diffuser and 30-s puffs every 30-s for the two small diffusers over 30 minutes
Sleep	Takeda et al. (2017)	Japanese cypress, Virginian cedarwood<br>cypress, pine oil blend	-	Fabric - pillow wrapped a towel with essential oils.<br>A range of 2 - 5 drops (0.1–0.25ml)
		True lavender		
		True lavender - sweet orange		
	Henry et al. (1994)	Lavender	-	Inhalation - electric fan<br><br>The amount of essential oil varied: two drops (one at 10 pm & one at 3 am) in day 1; four drops at the same time in day 2; three drops in day 3 and subsequent nights
Cognition	Jimbo et al. (2009)	Rosemary - lemon	A mixture of 0.04 ml lemon and 0.08 ml rosemary essential oil	Inhalation - electric fan
		Lavender - orange	A mixture of 0.08 mL lavender and 0.04 mL orange essential	
